# Detection of 12.5% and 25% Salt Reduction in Bread in a Remote Indigenous Australian Community

**DOI:** 10.3390/nu8030169

**Published:** 2016-03-16

**Authors:** Emma McMahon, Rozlynne Clarke, Rachael Jaenke, Julie Brimblecombe

**Affiliations:** 1 Wellbeing and Chronic Disease Division, Menzies School of Health Research, John Mathews Building, Royal Darwin Hospital Campus, Rocklands Dr, Darwin NT 0810, Australia; Rachael.Jaenke@menzies.edu.au (R.J.); Julie.Brimblecombe@menzies.edu.au (J.B.); 2 Division of Health Sciences, University of South Australia, 101 Currie St, Adelaide SA 5001, Australia; 3 Goodman Fielder, 39 Delhi Rd, North Ryde NSW 2113, Australia; Rozlynne.Clarke@goodmanfielder.com.au

**Keywords:** salt, reformulation, Indigenous Australian consumers, bread, acceptance, detection

## Abstract

Food reformulation is an important strategy to reduce the excess salt intake observed in remote Indigenous Australia. We aimed to examine whether 12.5% and 25% salt reduction in bread is detectable, and, if so, whether acceptability is changed, in a sample of adults living in a remote Indigenous community in the Northern Territory of Australia. Convenience samples were recruited for testing of reduced-salt (300 and 350 mg Na/100 g) *versus* Standard (~400 mg Na/100 g) white and wholemeal breads (*n* = 62 for white; *n* = 72 for wholemeal). Triangle testing was used to examine whether participants could detect a difference between the breads. Liking of each bread was also measured; standard consumer acceptability questionnaires were modified to maximise cultural appropriateness and understanding. Participants were unable to detect a difference between Standard and reduced-salt breads (all *p* values > 0.05 when analysed using binomial probability). Further, as expected, liking of the breads was not changed with salt reduction (all *p* values > 0.05 when analysed using ANOVA). Reducing salt in products commonly purchased in remote Indigenous communities has potential as an equitable, cost-effective and sustainable strategy to reduce population salt intake and reduce risk of chronic disease, without the barriers associated with strategies that require individual behaviour change.

## 1. Introduction

Reducing sodium (salt) intake has been identified as one of the most cost-effective interventions to improve population health [1]. The World Health Organisation recommends a maximum sodium intake of 2000 mg per day (equivalent to 5 g of salt/day) for adults [2]. It is estimated that globally 2.5 million deaths could be prevented each year if population salt intakes were reduced to the recommended level [1].

Many research trials have found that short-term adherence to a low salt intake is achievable in a highly-controlled research setting [3,4], however difficulty has arisen in translating these findings to achieving sustained population-level salt reduction [1]. Such interventions often involve considerable resources through intensive tailored dietary education and/or provision of pre-prepared meals, which are not feasible to implement at the population level. As approximately three-quarters of the salt consumed in Australia and other Westernised countries comes from processed foods [1,5], product reformulation is a viable option to reduce population salt intake [1,5,6]. The United Kingdom (UK) achieved a reduction in population salt intake from 9.5 g (3700 mg Na) in 2001 to 8.5 g (3100 mg) in 2011 through implementation of a UK-wide salt reduction strategy, part of which was setting voluntary sodium targets for numerous food products [7].

Bread is one of the biggest contributors of dietary salt in developed Western countries, including Australia, providing about 20%–25% of total salt intake [8]. The “Food and Health Dialogue” program launched by the Australian Federal Government in 2009 set a target maximum sodium content for bread of 400 mg/100 g [9]. Trevana *et al*. (2014) found that mean sodium content of bread in Australia fell significantly following the launch of this voluntary target from 454 mg/100 g in 2010 to 415 mg/100 g in 2013, however indicated that lower targets may be needed to achieve comparable success to that achieved in the UK [10].

Cardiovascular disease is the leading cause of mortality in Indigenous Australians [11]. Chronic kidney disease rates are growing rapidly; the prevalence of Indigenous Australians starting end-stage renal disease treatment has more than doubled in the last 10 years [12]. Approximately 30% of Indigenous Australians have high blood pressure [13], a strong risk factor for cardiovascular and chronic kidney diseases [14]. Therefore lowering salt intake in this population may be of considerable benefit for reducing blood pressure and risk of chronic disease [15,16]. Recently, the National Aboriginal and Torres Strait Islander Nutrition and Physical Activity survey indicated that average sodium intake of the Indigenous Australian population, not counting discretionary (table) salt, was 2400 mg/day [17]. All age groups had an average sodium intake above the recommended upper limit [17,18]. Even without including discretionary salt intakes in the estimates, children aged 2–3 and 4–8 years had average sodium intakes of 170% and 160% of the maximum recommended intake respectively [17,18]. We recently estimated total sodium (including discretionary salt) intake from store sales data in 20 remote Indigenous communities and found that estimated average per capita sodium was 2800 mg/day, far above the recommended amount [19].

Brimblecombe *et al*. (2013) previously used optimisation modelling to examine dietary change needed to achieve nutrient requirements in remote Indigenous communities without increasing cost [20]. A significant finding was that it was not possible to achieve below the recommended upper limit of salt intake with the current food supply [20]. Despite large shifts in dietary intake from processed to unprocessed food, the modelled diet provided 150% of the upper limit for salt intake, highlighting that changes within food manufacturing are essential to reduce intake to within recommendations [20].

The second biggest contributor of sodium in the Indigenous Australian population, after discretionary salt, is bread [19], therefore reducing the amount of salt in bread could reduce the salt intake of this population without requiring individual behaviour change. Bush Oven Outback Bread™ (Bush Oven) is a leading brand of bread primarily distributed to remote Indigenous communities across Australia. Our aim was: (1) to examine if the target consumer could detect a difference in salt levels between the breads; and (2) to assess liking of the reduced salt breads against the regular sodium version.

## 2. Materials and Methods

### 2.1. Reduced Sodium Breads

Bush Oven white and wholemeal breads were reformulated to reduce their sodium content, with two reduced sodium versions developed for each. Bush Oven High Fibre white bread was reformulated to reduce sodium from the current sodium content of 400 mg Na/100 g (Standard bread) to 350 mg Na/100 g and 300 mg Na/100 g. Bush Oven wholemeal bread was reformulated from current sodium content of 400 mg/100 g (Standard) to 350 mg Na/100 g and 300 mg Na/100 g. Prior to the present study, Goodman Fielder conducted internal quality testing, including: (i) microbial and shelf-life testing; and (ii) sensory discrimination and acceptability testing by an internal consumer panel.

### 2.2. Setting and Participants

This study was conducted in a large remote Indigenous Australian community in the top-end of the Northern Territory, Australia. Ethical approval was granted by the Human Research Ethics Committee of NT Department of Health and Menzies School of Health Research (white Bread: HREC-2014-2186, wholemeal bread: HREC-2015-2338).

Participants who were aged over 18 years, not pregnant and bread consumers were eligible for inclusion. Due to the high prevalence of smoking in the remote Indigenous population [17], it was not considered appropriate to exclude current smokers from the study. Participants were recruited from the community via word of mouth, handing out study flyers to different organisations and through making announcements at community meetings and over the community megaphone. Local Aboriginal research assistants were employed for the study to assist with recruitment and data collection. Upon completion of testing, participants were provided a pre-paid electricity card of $20 value as a thank you for their time.

### 2.3. Development of Questionnaires

A Yolgnu language is spoken as the first language by many in the participating community, with varying levels of English fluency, reading comprehension and writing abilities. To maximise validity and cultural appropriateness, standard questionnaires used for consumer testing were modified. The difference testing form required little modification; visual cues were added to maximise understanding. The questionnaire used for acceptability testing was modified to include plain language and visual cues, and to ensure appropriateness of characteristics tested. During development of the acceptability questionnaire advice and feedback was sought from Aboriginal Health Practitioners and community members. Aboriginal Health Practitioners (*n* = 3) with extensive experience working with remote Indigenous communities were consulted to assist with picture and language choice. Community members (*n* = 4) were consulted: (1) to determine the characteristics of white bread that they consider to be desirable (to ensure that these are captured when assessing liking of the bread); and (2) to provide feedback on comprehensibility of questionnaire elements.

Further consultation was sought when amending the questionnaires for the wholemeal bread testing. It was recommended to change the scale as the visual analogue scale used in white bread testing may not be well understood by some participants. Therefore a modified Likert scale in both the local language and English (see Section 2.4.2) was used for wholemeal bread testing. Community members provided advice about Likert scale options and assistance with translation.

### 2.4. Data Collection

Testing was carried out at a community centre over two days in June 2014 for white bread testing and three days in June 2015 for wholemeal bread testing. Prior to testing, bread loaves were sliced and the crust ends plus the second slice from each end were discarded. Each bread type was assigned a 3-digit code (with multiple codes for each of the bread type and test type).

Participants were explained the purpose of the study and given instructions in the local language or English at the beginning of testing. Participants were then seated and provided with a bottle of cold water to cleanse the palate between bread samples. During testing, an Aboriginal research assistant or an investigator assisted each participant by giving instructions and in some cases scribing for the participant. Investigators and Aboriginal research assistants were blinded to bread coding.

#### 2.4.1. Difference Testing

Triangle tests [21] were used to determine whether participants were able to detect a difference between Standard and salt reduced (350 and 300 mg Na/100 g) breads. The lower sodium bread (300 mg/100 g) *versus* Standard was tested first followed by the 350 mg Na/100 g *versus* Standard bread. Immediately prior to testing, crusts were removed from the bread samples, and slices were cut into halves. Order of presentation was randomised, and balanced, across the group to prevent order bias [22]. Each participant received a paper plate with three pieces of bread. The corresponding three-digit code for each sample was labelled on the plate (multiple codes were used for each bread to ensure participants had different coding than those they were seated near). Respondents were informed that two pieces of bread were the same and one was different and asked to identify the different sample by tasting left to right then circling the corresponding code of the different sample on the form provided. If they were unsure, participants were asked to guess (*i.e.*, forced choice method) [21]. Bottled water was supplied as a palate cleanser between samples.

#### 2.4.2. Acceptability Testing

Liking of breads were examined using acceptability testing [21]. The following process occurred for both white and wholemeal acceptability testing. Bread samples were prepared by slicing the loaves and discarding the crust ends plus the second slice from each end. Immediately prior to testing, bread slices were cut into halves. Each participant received a paper plate with one half piece of each of the three breads (Standard, 300 and 350 mg Na/100 g), presented in a random and balanced order (simultaneous presentation method [22]). Breads were coded by a 3-digit code; differing codes were used than those used for difference testing and multiple codes were used for each bread type (to ensure participants had different coding than those they were seated near). Participants were asked to taste breads from left to right, taking a drink of water between each sample, and rate their liking of the appearance, whiteness (for white bread only), colour (wholemeal bread only), flavour, sweetness, saltiness, texture, softness and overall liking.

**Figure 1:**
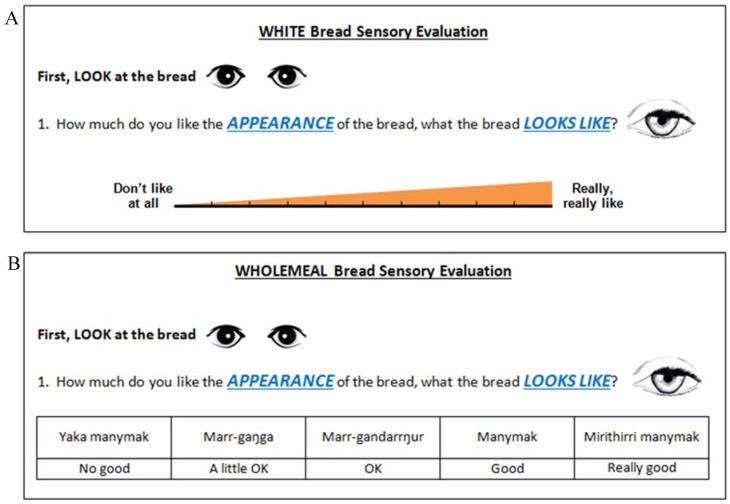
Scales used in white (**A**) and wholemeal (**B**) acceptability testing.

As discussed in Section 2.3, the rating scale used for white bread testing was modified for wholemeal bread testing. White bread testing participants rated their liking on a 10 mm visual analogue scale (Figure 1). For wholemeal bread testing, participants rated their liking on a 5-point Likert scale (Figure 1) with options written in English and in the local language. Options were “No good” (Yaka manymak), “A little ok” (Marr-gaŋga), “Ok” (Marr-gandarrŋur), “Good” (Manymak) and “Really good” (Mirithirri manymak).

### 2.5. Statistical Analysis

Difference testing was analysed using binomial probability testing (where probability of success is 0.33). Upper critical value was calculated (maximum number of correct identifications before statistical significance is reached) with significance set at 0.05. Difference in the number of correct identifications between smokers and non-smokers, males and females and across age groups was analysed using Chi square.

For white bread acceptability testing, ratings on the 10 mm visual analogue scale were measured with a ruler and recorded in centimetres (rounded to the nearest centimetre). For wholemeal bread testing, numerical values were assigned to the options from 1 (No good) to 5 (Really good). Acceptability testing was analysed using within subjects repeated measures two-way ANOVA (fixed factor for sample type and random factor for individual).

Data were analysed using Stata 14 (StataCorp LP, College Station, TX, USA). Significance level was set at *p* < 0.05.

## 3. Results

A total of 62 and 72 participants completed the white and wholemeal bread testing, respectively. Participant demographics are shown in Table 1.

In the white bread difference test >27/62 correct identifications of the different bread were required to be able to detect a significant difference. Results showed that 19/62 participants correctly identified the different sample in the 300 mg Na/100 g *versus* Standard and 26/62 in the 350 mg Na/100 g *versus* Standard, indicating that the difference was not detectable. Given there were 72 participants in the wholemeal bread test, >31/72 correct responses were required to establish a significant difference. As 25/72 participants correctly identified the different bread in both 300 mg Na/100 g *versus* Standard and 350 mg Na/100 g *versus* Standard tests, no significant difference was detected between the bread samples for both tests.

There was no significant difference in the proportion of smokers *versus* non-smokers who correctly identified the different bread in any of the difference tests (Table 2). There was a significant difference between males and females in correct identification of the different bread in 300 mg Na/100 g *versus* Standard white bread difference test, however this difference was not observed in 350 mg Na/100 g *versus* Standard white bread difference test nor in the wholemeal bread difference tests (Table 2). There were no significant differences in the proportion of correct identifications across age groups in wholemeal or white difference tests (all *p* values > 0.05).

**Figure 2:**
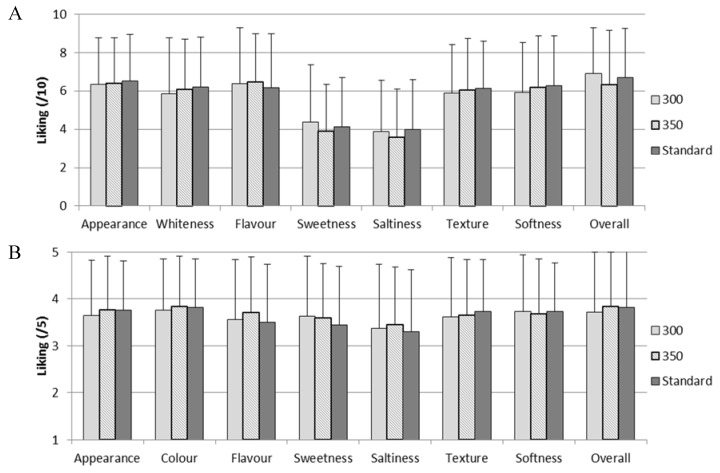
Average liking of Standard *versus* reduced sodium white (**A**) and wholemeal (**B**) breads. Error bars indicate standard deviation.

As no difference was detected, it is expected that acceptability would not differ between the breads. As expected, there was no significant difference in overall liking between Standard and reduced sodium (300 or 350 mg Na/100 g) white or wholemeal breads (all *p* values > 0.05), nor were there significant differences in liking of appearance, whiteness/colour, flavour, sweetness, saltiness, texture, softness or overall liking (all *p* values > 0.05; Figure 2). 

## 4. Discussion

We investigated whether 12.5% and 25% salt reduction in white and wholemeal Bush Oven bread was detectable by, or affected liking to, a sample of adults living in a remote Indigenous Australian community. To the authors’ knowledge, this is the first study examining consumer acceptability of a reformulated product targeting the Indigenous Australian population. We found that up to 25% salt reduction in bread (from approximately 400 to 300 mg Na/100 g) was not detected. As would be expected where a difference is not detected, liking was also not affected.

Our findings are consistent with studies conducted in other populations [23]. Jaenke *et al*. (2015) conducted a meta-analysis examining consumer acceptability of reduced salt products and found that when studies were pooled where sodium in bread was reduced by <40% (range 25%–37%), there was no significant change in consumer acceptability score (*p* = 0.13) [23]. However, significant reduction was seen when studies with larger reductions (<40%) were pooled [23]. Girgis *et al*. (2003) examined whether incremental changes to the salt content of bread reduced consumer acceptance in a sample of 110 staff in an Australian Hospital [24]. When sodium content was reduced from a baseline content of 780 mg/100 g by 5% per week for 5 weeks (~80 mg Na/100 g reduction per week) giving a final content of 585 mg Na/100 g, no significant change in liking was observed [24]; Most Australian breads are now lower in sodium than those tested by Girgis *et al*. [10,24]. Average sodium content of bread in Australia (415 mg Na/100 g) is above the target set by the Food and Health Dialogue (aiming for all breads to be lower than 400 mg Na/100 g) [10]. The current salt target for bread in the United Kingdom is an average sodium content of 360 mg/100 g by 2017 [25]. There is definite potential for further reductions of salt in Australian breads.

The relationship between salt intake and blood pressure is dose responsive and varies greatly between individuals [26]. It has been estimated that a reduction in salt intake as small as 0.5g (200 mg sodium) per day could have significant outcomes in terms of reduced cardiovascular mortality at the population level [7]. A randomised crossover trial in Argentina measured the efficacy of providing 30% reduced salt bread to 58 participants with high systolic blood pressure (BP) for reducing salt intake and BP [27]. Provision of reduced salt bread reduced average salt intake by 575 mg/day (95% CI 159 to 1000; *p* = 0.009), equivalent to 1.5 g salt, and reduced systolic/diastolic BP by 1.7/0.8 mm Hg (95% CI −2.8 to −0.5/−1.4 to −0.1, *p* < 0.05) [27]. Participants in this study consumed a considerable amount of sodium through bread (~950 mg Na/day), partially due to the high sodium concentration of bread in Argentina (average of 780 mg Na/100 g), therefore the impact seen in this study may not be transferable to populations where bread contributes less sodium to the diet [27]. Nevertheless this study demonstrates that it is feasible to develop a lower-salt bread that is acceptable to consumers and that can reduce salt intake by a level that can impact cardiovascular risk.

We previously found that bread contributes 18% of dietary sodium in remote Indigenous communities, ranging from 13% to 25% within individual communities. This equates to approximately 510 mg sodium per day coming from bread (range 360–690 mg/day) [19]. If all bread was reformulated to reduce sodium by 25%, estimated average sodium intake of those living in remote Indigenous communities would be reduced by 124 mg/day (range within communities 90–173 mg/day), equivalent to 0.3 g salt per day [19]. While there is no data to indicate that this magnitude of salt reduction would be clinically significant at the population level, individuals within the population who are hypertensive and/or who consume a lot of bread may benefit, without requiring individual behaviour change and without posing risk of adverse effect to others.

It is unlikely that reductions in sodium intake by bread reformulation would be compensated for by individuals increasing sodium intake elsewhere in the diet [28]. In a study conducted among 116 participants in the Netherlands, it was found that when salt was reduced in bread and served with a buffet style breakfast alongside a variety of fillings, participants did not compensate for the reduced salt content by choosing saltier fillings [28]. This resulted in sodium intake for the meal being reduced by an average of 230 mg/day (95% CI = 147 to 315 mg Na/day), equivalent to 0.6g salt/day, with 52% reduced salt bread compared with regular salt bread. Another recent study in the Netherlands tested the effects of serving a lunch comprised of reduced sodium products (29% to 61% sodium reduced bread, cold cuts, cheese, sandwich fillings, hot snacks, soups and salad dressings served buffet style) in a real life canteen setting and found that mean daily sodium intake was reduced by 1093 mg per day (95% CI 901–1285), equivalent to 2.5 g salt, compared with control [29]. This demonstrates how widespread reformulation to reduce salt content of processed foods could have a clinically significant impact on salt intake. Our group recently modelled the potential effects of wide-spread salt reduction in processed foods where salt is added during processing on population-level salt intake in remote Indigenous communities. We found that 38% salt reduction could reduce estimated average salt intake of this population to within dietary recommendations; whereas reducing salt content of bread alone, although important, would not reach dietary recommendations [19].

Compared to the wider Indigenous Australian population living in very remote Australia (hereafter referred to as “wider population” [30]), there was a lower proportion of males in the wholemeal bread testing (38% *versus* 48% in wider population), but a comparable gender distribution in the white bread testing (52% male). There was no difference in the proportion of males *versus* females who correctly identified the different bread in wholemeal bread testing, therefore this difference is unlikely to reduce the generalisability of the results. There was a greater proportion of participants in the oldest age group (21% in the 56+ age group for both white and wholemeal compared to 14% in the wider population) and less in the youngest age group (32% and 36% for white and wholemeal testing compared to 47% in wider population), however as there was no relationship between age and detection of the different bread in any of the difference tests, this is unlikely to affect the generalisability of the results. In the white bread testing, a small proportion (5%) of participants did not identify as Indigenous Australians, however these were not excluded from the analysis as the Bush Oven bread is available for purchase by both Indigenous and non-Indigenous Australians. Approximately 5% of people living in very remote parts of Australia identify as being a non-Indigenous Australian [30].

Potential limitations of the present study include including only one community, testing difference and liking in the same session, inclusion of smokers, provision of incentives, testing in adults only and the language barrier. Participants who smoke are often excluded from tasting studies due to the effects of smoking on taste and olfactory dysfunction [31]. However as an estimated 50% of the Indigenous Australian population who live in remote areas are current daily smokers [32], excluding smokers would have been impractical, and may have reduced the representativeness of the findings. There was no significant difference in the proportion of participants who could correctly identify the different bread between current smokers and current non-smokers, suggesting that inclusion of current smokers did not affect the findings. A further limitation is testing acceptability immediately following difference testing, as it was not feasible to have participants return for a second session, especially given limited time in the community. Provision of prepaid electricity cards to participants was intended as an acknowledgement of their time, however it acted as an incentive for some to complete the study. We believe this strengthened the study, rather than acting as a limitation, through increasing response rate and representativeness of the sample. Much consideration was given to reduce the language barrier as a limitation. Consultation as outlined in Section 2.3 aimed to ensure that the language and pictures used in questionnaires were appropriate. We employed Aboriginal research assistants to translate and assist participants with completing questionnaires. Investigators also assisted with questionnaires when all Aboriginal research assistants were busy. The difference testing was well understood by participants. There was, however, some feedback regarding the visual analogue scale following the white bread testing; the Aboriginal research assistants indicated that participants may have difficulty conceptualising their liking of the bread on a graduating line (Figure 1). While this is a potential limitation of the white bread acceptability testing, this is likely to have equally affected the results of all breads tested. Further, the results of white bread acceptability testing are consistent with those of the white bread difference testing; since participants did not detect a difference with salt reduction, it is unlikely that liking would change. Therefore, we believe that the results are accurate. Regardless, we revised the rating scale used for the wholemeal bread testing to a Likert scale with response options written in the local language as well as English and found that this was well accepted by participants. The Likert scale is not considered “gold standard” for measuring continuous variables (such as liking), but we found it to be far more suitable than a visual analogue scale in this population. This demonstrates how taking a pragmatic approach to research can be valuable in the remote Indigenous population. We recommend that those who wish to include the remote Indigenous Australian population in sensory research should take a pragmatic approach, should employ translators and/or local people to assist with data collection and should ensure that all aspects of the questionnaires are pre-tested.

## 5. Conclusions

In conclusion, we found that 25% salt reduction in wholemeal and white breads was not detectable to a sample of adults living in a remote Indigenous Australian community, nor did salt reduction affect liking of the breads. Salt reduction of all bread purchased in remote Indigenous communities could reduce population salt intake by an average of approximately 0.3 g/day. Reducing the salt content of products commonly purchased in remote Indigenous communities has potential as an equitable, cost-effective and sustainable strategy to reduce population salt intake and reduce risk of chronic disease, without the barriers associated with strategies that require individual behaviour change. We urge food manufacturers to consider the potential benefits of reducing salt content of their products for improving the health of their consumers.

## Figures and Tables

**Table 1:** Participant demographics.

Demographic	White Bread Testing	Wholemeal Bread Testing
Male	52% (*n* = 32/62)	38% (*n* = 27/72)
Indigenous	90% (*n* = 56/62)	100% (*n* = 72/72)
Age group:		
18–34 years	32% (*n* = 20/62)	36% (*n* = 26/72)
35–55 years	47% (*n* = 29/62)	42% (*n* = 30/72)
56+ years	21% (*n* = 13/62)	21% (*n* = 15/72)
Missing data	0% (*n* = 0/62)	1% (*n* = 1/72)
Smoking status:		
Non-smoker	45% (*n* = 28/62)	31% (*n* = 22/72)
Smoker	55% (*n* = 34/62)	64% (*n* = 46/72)
Missing data	0% (*n* = 0/62)	6% (*n* = 4/72)

**Table 2:** Difference testing results.

Test	All Participants		Gender (% Correct)			Smoking Status (% Correct)		
	Correct	*p* *	Male	Female	*p* ^†^	Smoker	Non-Smoker	*p* ^†^
White								
300 *vs.* Std	30% (19/62)	0.69	44% (14/32)	17% (5/30)	0.02	26% (9/34)	35% (10/28)	0.43
350 *vs.* Std	42% (26/62)	0.17	44% (14/32)	40% (12/30)	0.76	44% (15/34)	39% (11/28)	0.70
Wholemeal								
300 *vs.* Std	35% (25/72)	0.80	41% (11/27)	31% (14/45)	0.41	30% (14/46)	45% (10/22)	0.23
350 *vs.* Std	35% (25/72)	0.80	33% (9/27)	36% (16/45)	0.85	37% (17/46)	32% (7/22)	0.68

Values are % correct identifications of the different bread (number of participants correct/total sample size). * Analysed using bionomial probability; † Analysed using Chi square. Std = standard bread.
